# Noise in operating theatres, is it safe?

**DOI:** 10.1007/s00402-024-05489-x

**Published:** 2024-08-06

**Authors:** Maliha Ayoola, Diego Agustín Abelleyra Lastoria, Laura Casey, Sara Dardak, Roshan Rupra, Caroline Blanca Hing, Sarah Radcliffe, Catherine Kellett

**Affiliations:** 1 St George’s University of London, London, SW17 0RE UK; 2 St George’s Hospital NHS Foundation Trust, London, UK; 3 Curload Consultants Limited, Consultants in Acoustics, Somerset, UK; 4 Mohammed Bin Rashid University of Medicine and Health Sciences, Dubai, United Arab Emirates

**Keywords:** Noise, Orthopaedics, Safety, Trauma

## Abstract

**Introduction:**

Noise-Induced Hearing Loss (NIHL) is a condition caused by repeated exposure to loud noise, with operating theatre personnel potentially at risk. The aims of this study were to establish the typical noise levels in orthopaedic theatres and to compare these to The Control of Noise at Work Regulations 2005.

**Materials and methods:**

We measured the average noise levels in 40 trauma and orthopaedic surgeries in a single centre. We used the Decibel X app to take measurements, then performed corrections to ascertain noise levels at the surgeon’s ear (L_eq_). The daily noise exposure level for theatre staff for each procedure (L_EP, d_) and the L_EP, d_ over an average 8-hour working day when performing different groups of procedures were calculated. Data were analysed using descriptive statistics, ANOVA, t-test and the Pearson coefficient of correlation.

**Results:**

The L_EP, d_ lower action value (80 dBA) as set by the Health and Safety Executive (HSE) was met by performing a single revision total knee replacement or a right open ankle debridement. Assuming three procedures are conducted per list, lists consisting of joint replacements (82 dBA) or medium elective procedures (81 dBA) exceed this lower limit. Additionally, lists comprising large and medium bone fractures would be within 1 dB of the limit (79 dBA and 79 dBA, respectively). Soft tissue (74 dBA), arthroscopic (73 dBA), and small bone fracture (71 dBA) procedures had the lowest L_EP, d_. The greatest contributors to noise levels were surgical instruments. The number of people in the room made a significant difference to noise levels (*p* = 0.032).

**Conclusions:**

We have established the baseline noise levels in various orthopaedic procedures. Measures should be taken to meet UK regulations. Further research should determine suitable measures for protection from hearing damage for theatre staff and evaluate the risks high noise levels pose to patients.

**Supplementary Information:**

The online version contains supplementary material available at 10.1007/s00402-024-05489-x.

## Introduction

Consistent exposure to loud noise can cause permanent damage to structures within the inner ear, resulting in hearing loss. Noise induced hearing loss is one of the most common causes of hearing loss worldwide [1]. Considering damage caused by noise is cumulative, individuals who are continually exposed to high noise levels are at an increased risk of developing NIHL [2]. In addition, excessive exposure to noise may lead to difficulties with communication, hypertension, and difficulty sleeping [3].

In adults, NIHL mainly develops due to noise in the workplace, with ten years of exposure to regular noise resulting in moderate to severe hearing loss [2]. Multiple regulations have been set in place to mitigate this [4–6]. Employers have a legal obligation to provide risk assessments of potentially high noise levels in the workplace and to reduce the noise exposure levels of the employees to the lowest reasonable and practicable level [6]. The Control of Noise at Work Regulations 2005 established a lower noise exposure action level of 80 dBA, and increased requirements on behalf of employers and employees, such as the requirement to provide regular health surveillance/audiometric testing for employees with upper action level noise exposure [7]. In addition, if the 80 dBA limit is exceeded, employers must perform a risk assessment and give staff training, as well as provide hearing protection on request. The absolute limit for noise exposure in the workplace as set by the HSE is 87 dBA, even if workers wear hearing protection [7].

There are no specific guidelines to reduce the risk of developing NIHL in trauma and orthopaedic surgery. Power tools such as saws and drills and occasional music are potential sources of noise, and may pose a risk of causing NIHL in operating theatre personnel [8]. Previous research is insufficient to establish safe noise levels in trauma and orthopaedic surgery. Studies have measured noise in multiple different specialties [9], or have evaluated a limited number of instruments [10] and procedures [10–12]. Therefore, this study has been set up as a pilot study, where the aims were to establish the typical noise levels in orthopaedic theatres, and to compare these to the recommended values for daily noise exposure levels set by UK Legislation. This could then be used to determine whether more research is necessary into this area and specific types of procedure.

## Methods

### Surgical procedures

A service evaluation of noise levels in trauma and orthopaedic theatres was conducted between January 2022 and May 2023 in a large tertiary trauma centre (Audit reference = AUDI002019). A total of 40 operations were recorded. These were performed across six different operating theatres. There were no restrictions placed on recordings based on time of day, surgical procedure, and length of procedure. The operations recorded were selected via convenience sampling. Measurements were taken by four different people who had been trained to use the noise measuring App. Theatre staff were unaware that noise measurements were taking place to avoid the Hawthorne effect (a phenomenon whereby the subjects of a study alter their normal behaviour if they are aware that they are being observed [13]).

Surgical procedures were categorized to calculate mean noise level exposure per group. These were joint replacement, long bone fractures, medium bone fractures, small bone fractures, arthroscopy, medium elective procedures, and soft tissue procedures. The joint replacements and long bone fracture groups tend to use more power tools, and we would anticipate higher noise levels from these procedures compared to soft tissue procedures which do not use such tools. These, or similar, groups could be used to determine which types of procedure could generate high noise levels and benefit from further study. Our study did not allow for detailed analysis of individual instruments use in each procedure, however our groupings have put together procedures which tended to use similar instruments.

### Noise recordings

The Decibel X Application was used across different cellular mobile devices [14]. This is a sound level meter application with adjustable settings, able to record sound pressure levels in A-weighted decibels. A-weighting was chosen as it mirrors the human ear’s response to noise [15]. Fast response time was used so sudden changes in noise levels could be detected. The application was used to measure the average (L_Aeq_), minimum (L_Amin_), and maximum (L_Amax_) noise levels of each surgery. Recordings were taken from a single point in each operating theatre throughout the operation. Distance to the operating table was recorded. Sources of noise that contributed to the overall noise levels were recorded. Recordings took place between surgical timeout and wound closure.

### Data processing

**Fig. 1:**
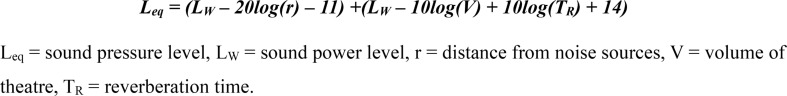
Equation used to calculate sound pressure at surgeon’s ear

To ascertain how noise levels can affect the hearing of the surgeons, calculations were carried out to correct for the fact that the measurements were not taken at the surgeons’ ear. Sound pressure must be calculated to yield an objective measurement of the sound level in a room [16]. This calculation considers the distance between the recording device and the noise sources, the room volume, the level of acoustic absorption in the room and the distance between the noise source and the surgeon. These can be used to measure the sound power emitted from a noise source, and the average noise level at the surgeon’s ear (L_eq_) (Fig. 1).

The noise exposure level for theatre staff for each procedure (L_EP, d_) was calculated taking into account procedure duration. The minimum, maximum, and mean L_EP, d_ for each surgery group were calculated.

Using mean procedure L_EP, d_ for each surgery group, the L_EP, d_ over an average 8-hour working day for each surgery group was calculated (assuming three procedures are performed per day on average, taking into account the duration of long procedures).

### Statistical analysis

The data were entered into IBM-SPSS for Windows version 29.0 [17]. Continuous variables were described using measures of central tendency and measures of dispersion. The Kolmogorov-Smirnov test was employed to assess the normality of continuous variables (Procedure LEP, d) in both the raw data and per procedure. T-tests and ANOVA were utilized to compare means among two groups or more than two groups, respectively. The Pearson coefficient of correlation was used to test the association between Procedure LEP, d, and the number of people present in the operation. A P-value of less than 0.05 was considered significant in all statistical analyses. As a decibel is a logarithmic ratio, the data were analysed accordingly.

## Results

### Procedures recorded

A total of 40 procedures were included. These included seven joint replacements, nine large fractures, nine medium fractures, two small fractures, three arthroscopies, two medium elective procedures, and eight soft tissue procedures (Table 1). Operation duration was not recorded in two procedures (removal of left ankle hexapod + cast application and left femoral shaft reconstruction). Therefore, L_EP, d_ for these could not be calculated.

### Noise levels recorded

The procedures were divided into groups for analysis: Joint Replacement, Long Bone Fractures, Medium Bone Fracture, Small Bone Fractures, Arthroscopic Procedures in the Knee, Medium Elective Procedures, and Soft Tissue Procedures (Table 1). The highest mean L_eq_ was recorded for Joint Replacement (77 dBA), followed by Medium Elective Procedures (76 dBA), Long Bone Fractures (74 dBA), Medium Bone Fractures (74 dBA), Soft Tissue Procedures (69 dBA), Arthroscopic Procedures in the Knee (68 dBA), and Small Bone Fractures (65 dBA).

When adjusting for procedure duration, the L_EP, d_ lower action value (80 dBA) was met by performing a single revision total knee replacement or an open ankle fracture debridement and ORIF. Assuming three procedures of the same category are performed in a day, a list consisting of joint replacements (82 dBA) or medium elective procedures (81 dBA) exceed this lower action value. Additionally, lists comprising long and medium bone fractures would be within 1 dB of the 80 dBA limit (79 dBA and 79 dBA, respectively). The operation duration for one left femoral shaft reconstruction (long bone fracture) was not recorded, and could not be included in the calculation of L_EP, d_ for its group.

Soft tissue procedures (74 dBA), arthroscopic procedures (73 dBA), and small bone fractures (71 dBA) had the lowest L_EP, d_ for three procedures amongst all categories. However, the L_EP, d_ for small bone fractures was calculated using data from a single right metacarpal open reduction and internal fixation (ORIF). This is because the operation duration was not recorded for a removal of a left ankle hexapod and subsequent cast application.

The greatest contributors to the noise levels were surgical instruments. The number of people in the room made a significant difference to the noise levels (*p* = 0.032). This may be because more people attend complicated and therefore “noisier” procedures. Interestingly the operations where music was played were significantly quieter overall (*p* = 0.045).

Joint replacement had significantly higher noise levels than all other groups of surgeries (ANOVA, p value range from 0.001 to 0.021), except medium elective procedures (*p* = 0.574).

## Discussion

Previous studies state there is insufficient evidence to reliably conclude that noise in trauma and orthopaedic operating theatres is sufficient to cause NIHL [8, 11, 18]. In our pilot study, we demonstrate noise levels surpass legislative guidelines, which could affect theatre staff. Theatre personnel may have multiple roles, including anaesthetists, nurses, radiographers, and surgeons. Only the surgeon, scrub nurse, and anaesthetist are present throughout the entire operation, with other staff able to leave the theatre if required. Therefore, the findings of this study are most applicable to these healthcare professionals. They are also the closest to the surgical tools, which contribute greatly to the noise levels in theatres [9].

Assuming an 8-hour working day in which three procedures are performed, the lower noise exposure action value outlined in the Control of Noise at Work Regulations 2005 [7] was exceeded in lists consisting of joint replacements and medium elective operations. Additionally, lists comprising long and medium bone fractures were within 1 dB of the noise action value. With these being shorter operations, more could be performed in a single day, potentially exceeding the 80 dBA limit. Therefore, health and safety guidelines specific to trauma and orthopaedic surgery are required for the prevention of NIHL in surgeons. According to the HSE, since the lower noise exposure action value was exceeded, employers have a duty to implement and offer measures to protect the personnel in the theatres [7]. This is not only limited to surgeons; whilst nurses and anaesthetists are positioned farther from the instruments, they may carry out more lists per week than a surgeon, and could therefore be at greater risk of NIHL.

Use of power tools such as saws and drills may lead to short bursts of high noise levels, with their cumulative effect resulting in permanent hearing loss [12]. Surgeons who are exposed to these brief high levels of noise for many years may suffer from this cumulative effect, with 10 years of consistent exposure to brief moments of loud noise demonstrated to cause hearing loss [2]. In a study by Willet [19], there was evidence of 50% of orthopaedic surgeons experiencing NIHL after over 22 years of work in trauma and orthopaedic surgery. This can lead to decreased quality of life, tinnitus, and noise-related physiological stress [9].

In addition to the risk posed to healthcare professionals, the noise levels in theatres can have a detrimental effect on the health and safety of patients. Patients are more vulnerable to hearing damage when anaesthetised [20]. Anaesthetic drugs relax the stapedius muscle, which is responsible for protecting the cochlea from loud noise [20]. The absence of this mechanism can make patients more susceptible to their effects, such as damage to hair cells in the inner ear [1]. The high noise levels identified in this study indicate patients’ wellbeing may be at risk. Since this was not among our aims, further research exploring the effect of noise on patients and evaluating modes of ear protection is required to test our hypothesis.

Reducing overall noise levels occurring during surgery may not be an appropriate strategy to mitigate the risk of NIHL. The number of people in the operating theatre had a statistically significant impact on noise levels. Further, reducing theatre personnel may come at the expense of sufficient human-power to efficiently carry the operation to completion. A reduction in conversation may not be feasible either, given the importance of communication in operations.

Interestingly, the operations in which music was played were significantly quieter overall (*p* = 0.045). This was an unexpected finding and may have occurred because music was predominantly played in quieter, less complex operations. Additionally, the level of music played in theatres is likely to be lower than in other workplaces such as engineering workshops, and thus contribute less to the overall procedure L_EP, d_. This might be for the comfort of the patient and to facilitate staff communication. Additionally, a recent survey found 73% of surgeons considered music decreased their anxiety and stress levels [21].

While instruments were the main source of high noise levels, manufacturers may be able to decrease the noise they emit [10, 18]. Communication and collaboration between hospitals and manufacturers to facilitate the development of quieter tools is required, in the interest of the safety of the theatre personnel. Further research regarding which instruments generate the loudest noise would be aid this process.

Though hearing protection could mitigate the effects of high noise levels, it may hinder communication between staff [8]. A pragmatic approach is therefore required, using current evidence to determine which operations necessitate hearing protection by taking into account noise levels generated. Tools developed by the HSE can be used to determine whether hearing protection is sufficient to protect against a specific noise level [22]. Since this could impair communication, further work should explore whether hearing protection use can be restricted to the parts of the procedure with high noise intensity. The provision of hearing protection with built-in communication systems could aid clear communication. However, this may be hindered by practical considerations of cost and infection control.

The limitations of this study must be taken into account when interpreting its results. Firstly, the accuracy of measurements could have been increased by using a professional sound level meter, such as a class 1 m operated by trained personnel [23]. However, given professional training in noise measurements is required, a mobile App was a suitable but albeit less reliable alternative [24]. Secondly, measurements were taken in a single centre, which may impact the generalisability of our results. The use of instruments provided by different manufacturers in other hospitals as well as varying room acoustics in other hospital theatres may lead to different noise levels being recorded. We grouped procedures into categories for ease of calculation of average noise levels and assessment of their cumulative effects. However, this approach may not be applicable to theatre lists comprising a variety of procedures. Finally a larger sample size would be required for further analysis assessing the occupational health risks due to noise in operating theatres. Similar results were also demonstrated in the review paper by Mistry et al., who also concluded that further studies were required [25].

**Table 1:** Noise levels recorded in trauma and orthopaedic procedures

Surgery	Average Noise Levels (dBA)	Leq at Surgeon’s ear	Number of People	Did Music Play?	Instruments Used	Duration (minutes)	Procedure LEP, d
Joint replacement							
1st stage revision total knee replacement	64.7	82.6	10	No	Mallet, Osteotomes, Diathermy, Saw	243	80
Total hip replacement	69.5	85.4	9	No	Diathermy, Mallet, Osteotome, Reamers	106	79
Patellofemoral joint replacement	64.2	82.1	8	No	Drill, Saw, mallet, reamers	138	77
Left total hip replacement	68	81.4	8	No	Drill, Saw, mallet, reamers	158	77
Revision total hip replacement	64.7	78.1	12	Yes	Diathermy, Mallet, Osteotomes, Reamers	263	76
Right total knee 1st stage revision	65.3	81.2	12	No	Powersaw, mallet	128	75
Total hip replacement	62.9	78.8	8	No	Drill, reamers, diathermy, mallet	155	74
*Mean LEP*,* d*							77.1
*LEP*,* d over three procedures*							82.1
Long bone fractures							
Left femoral shaft intramedullary nail	64.5	80.4	10	No	Screwdriver	169	76
Right tibial plateau ORIF	69.3	84.1	13	Yes	Drill, osteotome	53	75
Removal of left femoral nail	68.3	81.7	9	Yes	Drill	79	74
Tibial fracture ORIF	61.3	77.2	12	No	Drill	219	74
Right tibial plateau ORIF	67.5	82.3	12	Yes	Drill, osteotome	64	74
Left acetabulum ORIF	65.5	80.3	11	Yes	Drill, mallet	96	73
ORIF/percutanous fixation of left acetabulum	65.5	80.3	8	No	Drill, mallet	96	73
Bilateral ORIF tibia	66.2	79.6	7	No	Drill	73	71
Left femoral shaft reconstruction	69.1	85.0	Not recorded	Not recorded	Not recorded	Not recorded	Unable to calculate
*Mean LEP*,* d*							73.9
*LEP*,* d over three procedures*							78.9
Medium bone fractures							
Right open ankle fracture debridement and ORIF	66.9	84.8	10	No	Mallet, screwdriver	162	80
Distal radius ORIF	66.7	82.6	9	No	Drill, wire driver	123	77
Right distal humerus ORIF	63.1	77.9	10	Yes	Drill	195	74
Ilizarov frame application for pilon fracture	62.7	78.6	8	Yes	Drill, wire driver	160	74
Ankle ORIF	61	76.9	8	No	Drill, reduction forceps, diathermy	106	70
Wrist fracture ORIF	59.5	75.4	12	No	Drill, wire driver	102	69
Ankle ORIF	62.4	75.8	7	Yes	Drill, wire driver, reduction forceps, diathermy	85	68
Removal of metalwork	66.9	80.3	7	Yes	Screwdriver	20	67
Wrist ORIF	59.3	75.2	8	No	Drill, reduction forceps, diathermy	64	66
*Mean LEP*,* d*							74.1
*LEP*,* d over three procedures*							79.1
Small bone fractures							
Right metacarpal ORIF	66.1	73.5	9	Yes	Drill, scalpel, self-retaining retractor, plate, k-wires	75	65
Removal of left ankle hexapod + cast application	63.7	77.1	11	No	Drill	Not recorded	Unable to calculate
*Mean LEP*,* d*							65.5
*LEP*,* d over three procedures*							70.5
Arthroscopy							
Knee arthroscopy	63.3	79.2	8	No	Arthroscopy set	41	69
Knee arthroscopy and microfracture	60	75.9	8	No	Arthroscopy set	70	68
PCL reconstruction	64.4	80.3	8	No	Arthroscopy, drill, mallet	17	66
*Mean LEP*,* d*							67.5
*LEP*,* d over three procedures*							72.5
Medium elective procedures							
Trochleoplasty	62.2	80.1	8	No	Osteotomes, mallet, drill, wire driver	220	77
Epiphysiodesis of distal femur and proximal tibia	70.6	84.0	7	No	Scalpel, drill, mallet	54	75
*Mean LEP*,* d*							75.7
*LEP*,* d over three procedures*							80.7
Soft tissue procedures							
Bilateral gastrocnemius lengthening	66.9	80.3	7	No	Scalpel, forceps	105	74
MPFL reconstruction	63.9	79.8	8	No	Arthroscopy set, mallet,	94	73
Incision and drainage of abscess	65.2	78.6	8	Yes	Diathermy	60	70
Left hip steroid injection	69.1	85.0	10	Yes	Injection	12	69
Closed reduction of hip dislocation	62.7	78.6	7	No	None	33	67
Left ulnar collateral ligament repair	66.3	73.7	10	Yes	Forceps, Langenbeck retractors, scalpel	38	63
VAC change	63.2	76.6	6	No	VAC set	15	62
Trigger finger release	62.5	69.9	6	No	Dissecting scissors, scalpel, retractors	14	55
*Mean LEP*,* d*							69.3
*LEP*,* d over three procedures*							74.3

Key:

dBA: A-weighted decibels

Leq: Sound pressure at surgeon’s ear

LEP, d: Noise exposure level for theatre staff for each procedure

ORIF: Open reduction and internal fixation

PCL: Posterior cruciate ligament

MPFL: Medial patellofemoral ligament

VAC: Vacuum-assisted closure

## Conclusion

This study has shown that the 80 dBA lower action level set by UK legislation is exceeded when performing lists consisting of joint replacements and long bone fractures. Since this is a pilot study, we cannot explicitly state that noise in trauma and orthopaedic theatres is not safe. However, we believe that this study has shown that there is valid cause for concern and that a suitable assessment of the risk from noise during trauma and orthopaedic procedures must be undertaken. Measures should be taken to meet regulations. A pragmatic approach is therefore required, using current evidence to determine which operations necessitate hearing protection. Further research must evaluate the risk of high noise level exposure to patients.

In summary, noise in operating theatres, is it safe? Our pilot study has shown that the levels set out in the Control of Noise at Work Regulations 2005 can be exceeded, on the balance of probabilities, during certain high noise procedures. We therefore recommend that a formal study be completed to determine the risk to theatre staff and patients.

## Electronic supplementary material

Below is the link to the electronic supplementary material.

Supplementary Material 1

## Data Availability

Not applicable.
