# Correction: Noise in operating theatres, is it safe?

**DOI:** 10.1007/s00402-024-05747-y

**Published:** 2025-02-25

**Authors:** Maliha Ayoola, Diego Agustín Abelleyra Lastoria, Laura Casey, Sara Dardak, Roshan Rupra, Amar Khamis, Caroline Blanca Hing, Sarah Radcliffe, Catherine Kellett

**Affiliations:** 1 St George’s University of London, London, SW17 0RE UK; 2 St George’s Hospital NHS Foundation Trust, London, UK; 3 Curload Consultants Limited, Consultants in Acoustics, Somerset, UK; 4 Mohammed Bin Rashid University of Medicine and Health Sciences, Dubai, United Arab Emirates

**Archives of Orthopaedic and Trauma Surgery (2024) 144:3343–3349**

10.1007/s00402-024-05489-x

In the original version of this article, co-author Amar Khamis name was missing and author names should have read

Maliha Ayoola^1^, Diego Agustín Abelleyra Lastoria^1^, Laura Casey^2^, Sara Dardak^2^, Roshan Rupra^2^, Amar Khamis^4^, Caroline Blanca Hing^2^, Sarah Radcliffe^3^, Catherine Kellett^4^

