# Supplementary material for: Noise in operating theatres, is it safe?
Source: Arch Orthop Trauma Surg. 2024 Aug 6;144(8):3343–9. doi: 10.1007/s00402-024-05489-x (PMC11417073; doi:10.1007/s00402-024-05489-x)

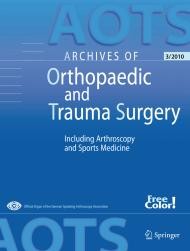

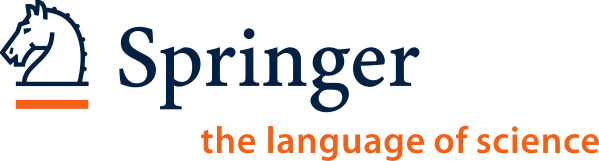


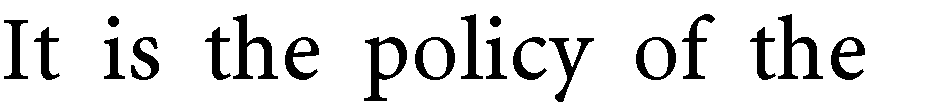

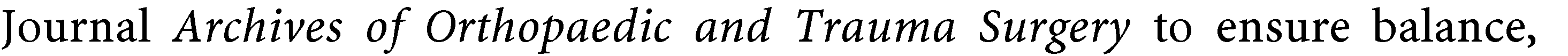

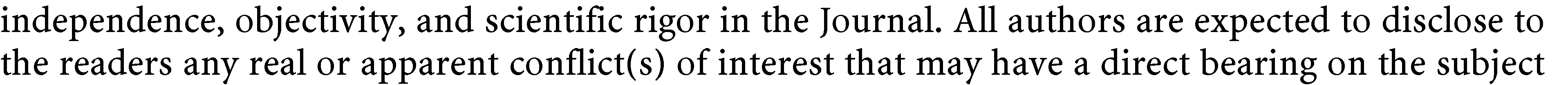

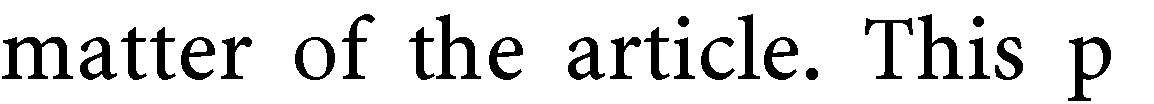

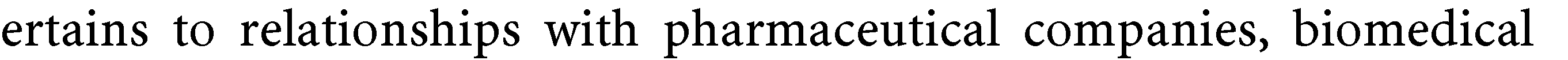

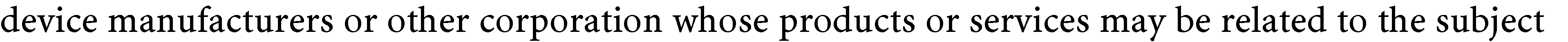

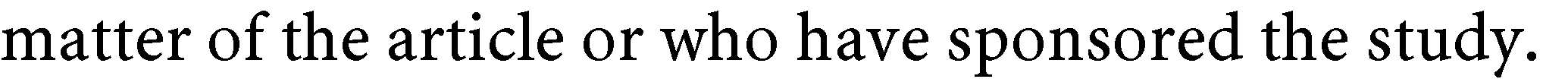

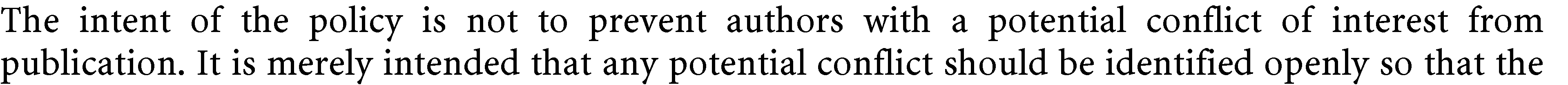

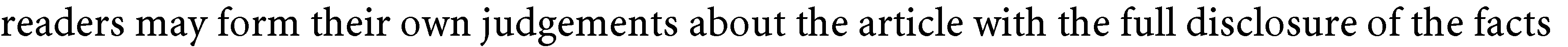

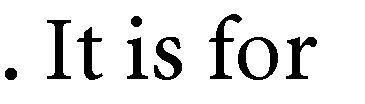

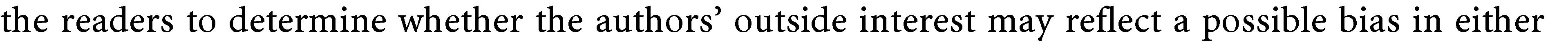

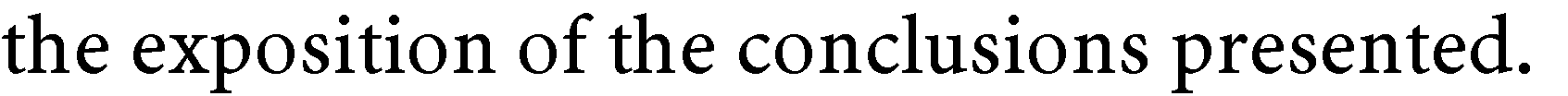

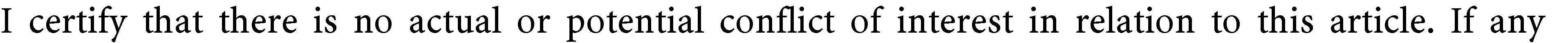

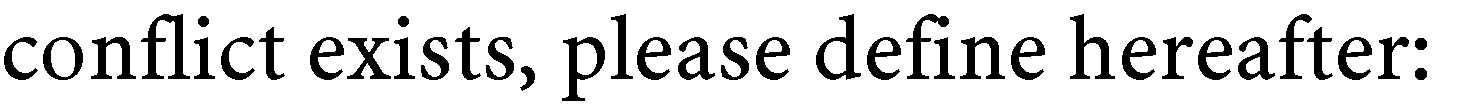

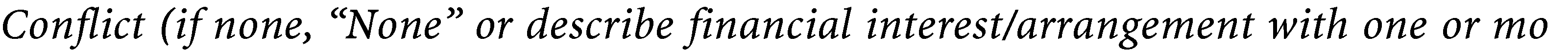

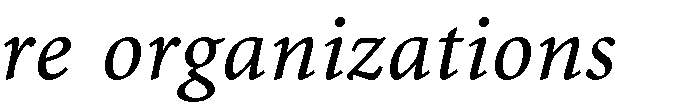

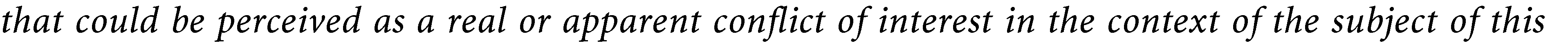

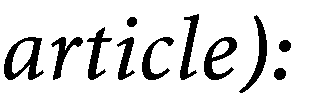

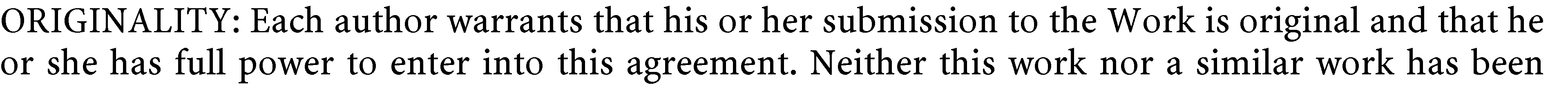

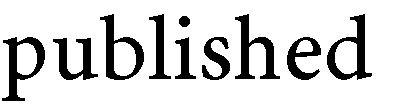

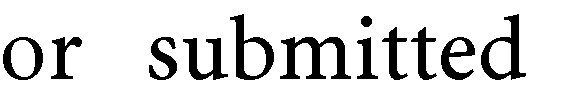

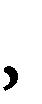

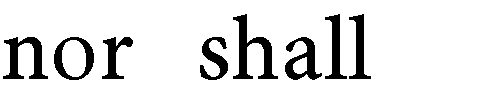

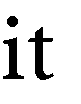

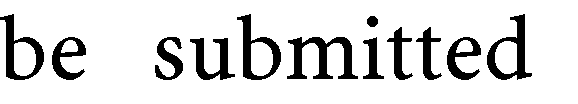

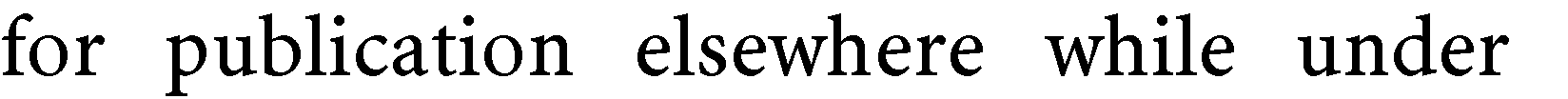

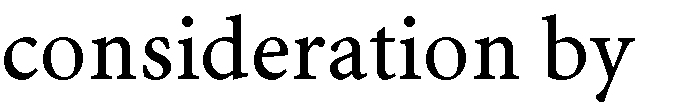

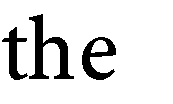

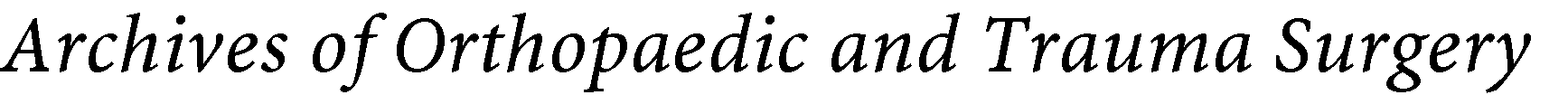

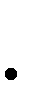

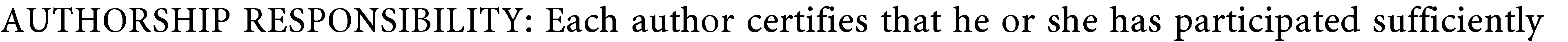

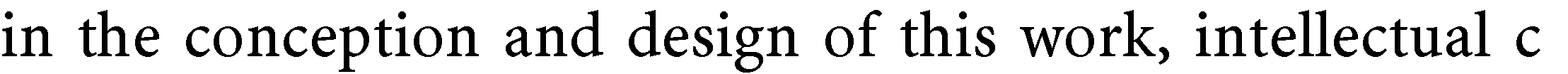

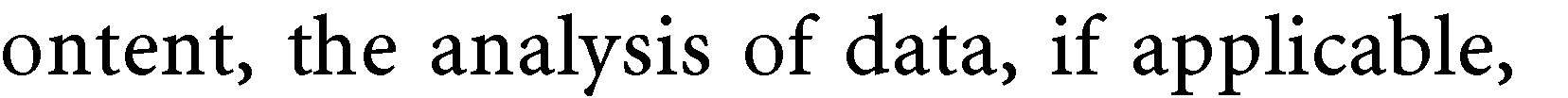

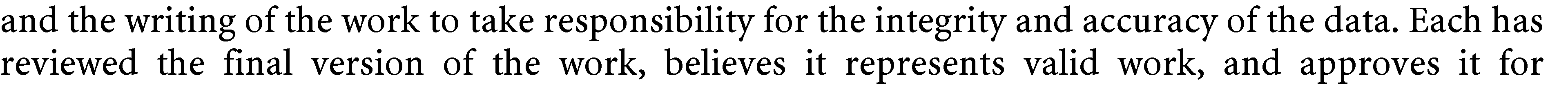

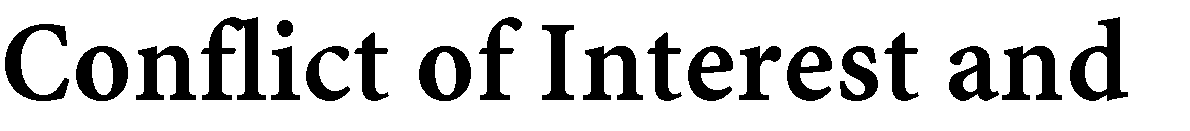

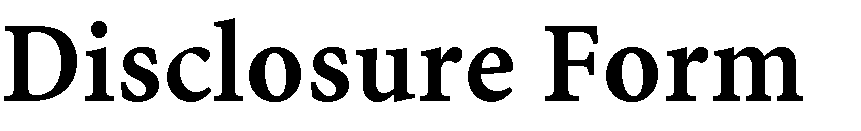


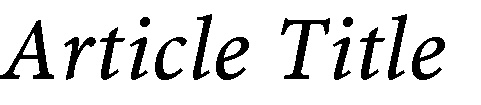

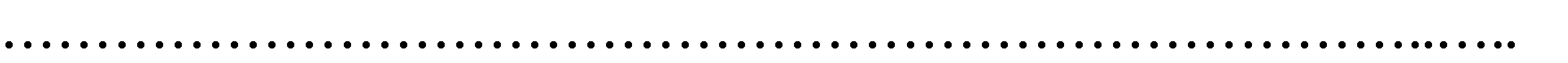

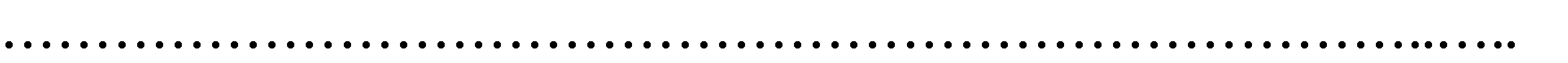

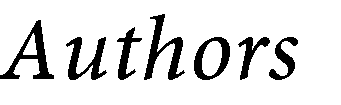

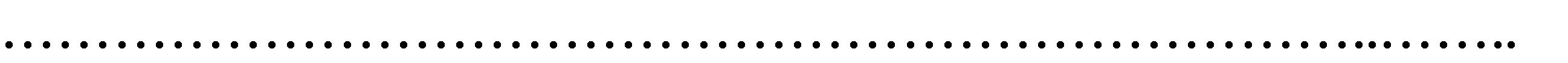


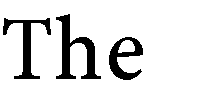

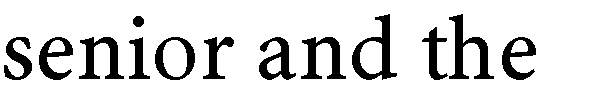

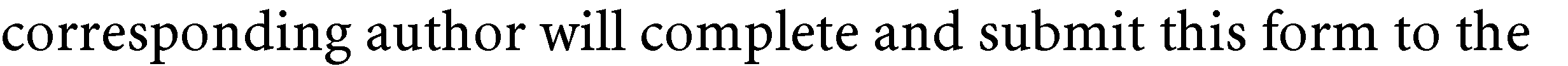

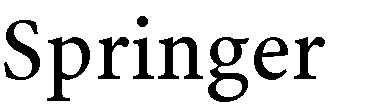

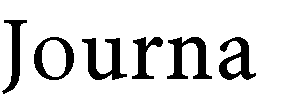

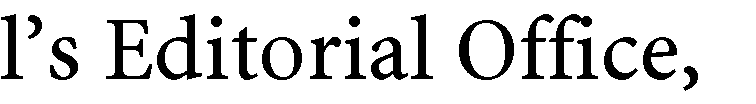

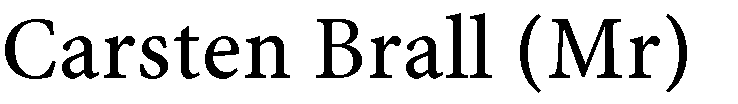

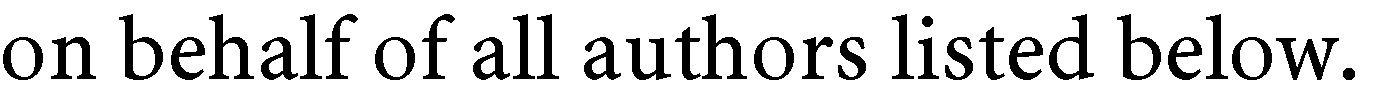

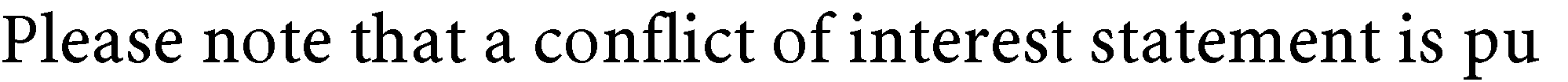

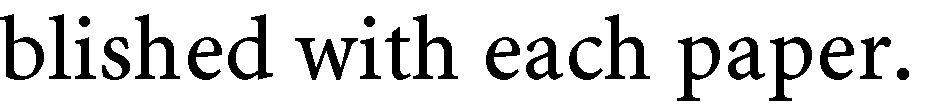


Maliha Ayoola, Diego Agustín Abelleyra Lastoria, Laura Casey, Sara Dardak, Roshan Rupra, Caroline Blanca Hing, Sarah Radcliffe, Catherine Kellett

Noise in operating theatres, is it safe?


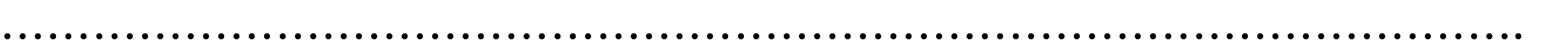

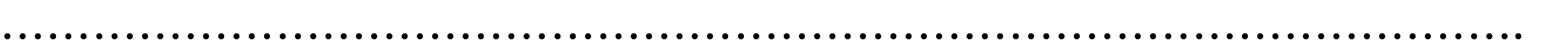

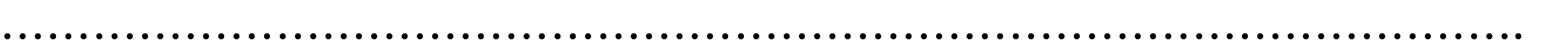


None


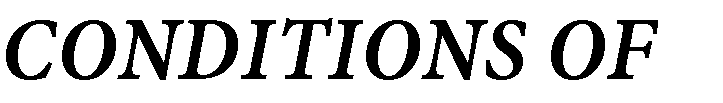

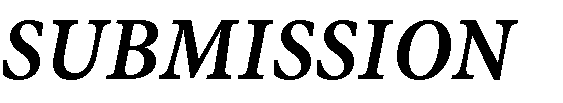

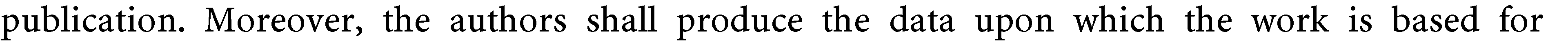

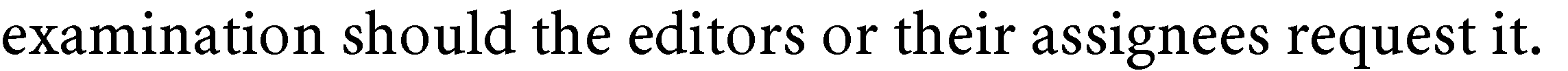


Catherine Kellett


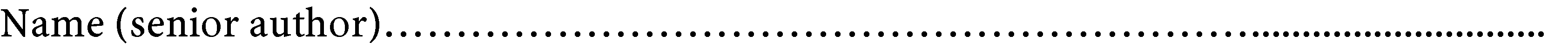


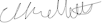


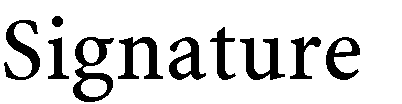

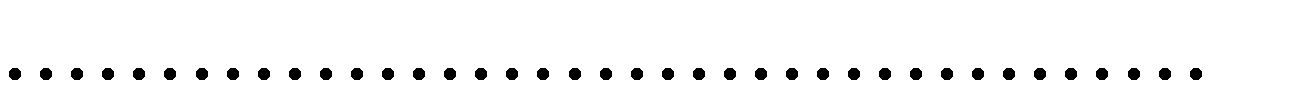

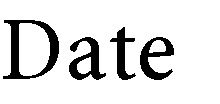


17/06/2024


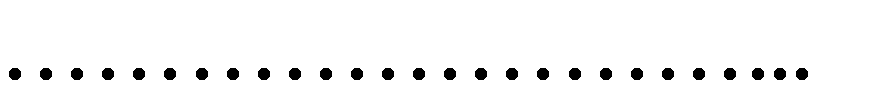

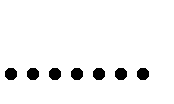


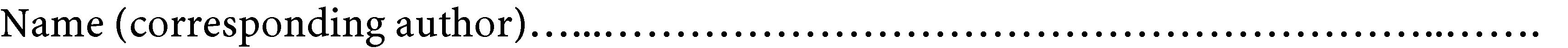


Diego Agustín Abelleyra Lastoria

17/06/2024


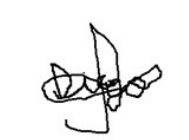

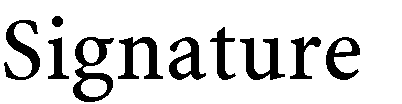

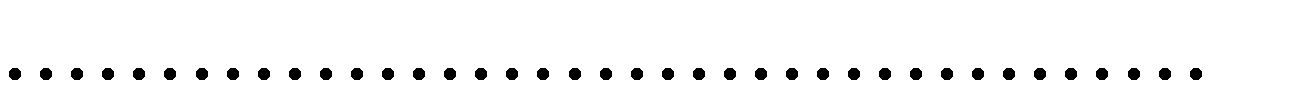

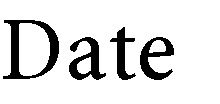

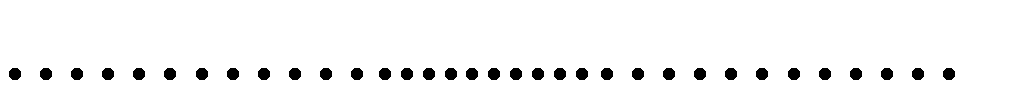


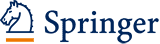


<http://www.springer.com/journal/402>


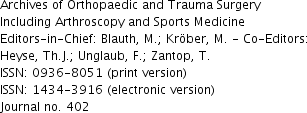

Supplement: Supplementary file 1 — Supplementary Material 1 [file 402_2024_5489_MOESM1_ESM.docx]
